# FOP: From Biomolecules to Hope

**DOI:** 10.3390/biom15030328

**Published:** 2025-02-24

**Authors:** Frederick S. Kaplan, Robert J. Pignolo

**Affiliations:** 1 Department of Orthopaedic Surgery, Perelman School of Medicine, University of Pennsylvania, Philadelphia, PA 19104, USA; 2 Department of Medicine, Perelman School of Medicine, University of Pennsylvania, Philadelphia, PA 19104, USA; 3 Center for Research in FOP and Related Disorders, Perelman School of Medicine, University of Pennsylvania, Philadelphia, PA 19104, USA; 4 Department of Medicine, Mayo Clinic College of Medicine, Mayo Clinic, Rochester, MN 55905, USA; pignolo.robert@mayo.edu

In the introduction to his 1970 textbook *BIOCHEMISTRY*, Albert Lehninger wrote “Living things are composed of lifeless molecules [1]”. More than half a century and worlds of insight later, we know a lot more about those lifeless molecules, the genes that encode them, the pathways they belong to, and the lifelong devastation that errors in those lifeless molecules may wreak. There may be no greater illustration of that than the second skeleton of heterotopic bone of the ultra-rare disorder fibrodysplasia ossificans progressiva (FOP) [2].

The mystery of FOP began to fade in 2006 with the discovery of the FOP gene—a transformative event that stimulated the development of genetically correct mouse models and novel therapeutics that fueled the advent of clinical trials [3,4].

Fibrodysplasia ossificans progressiva (FOP), a disorder of congenital skeletal malformations and progressive extraskeletal ossification, is the most severe form of heterotopic ossification (HO) in humans [2]. Gain-of-function mutations in Activin A Receptor Type I (ACVR1), a bone morphogenetic protein (BMP) type 1 receptor, cause FOP by dramatically altering the normal physiologic functions of ACVR1, impacting BMP signaling as well as interacting pathways [3,5,6,7]. Gain-of-function mutations in ACVR1 emerge as the ultimate origin of all FOP phenotypes [8].

A father of a young child with FOP asked of us, “How will the discovery of the gene help my child?” The answer is clear. The FOP gene discovery means hope. The FOP gene discovery enabled the development of animal models of FOP, which have been instrumental in testing novel therapeutics for druggable targets [4]. Animal models validated the dysregulated BMP signaling pathway in FOP and opened the door to understanding the pathophysiology of heterotopic ossification (HO) in FOP as well as the discovery of the progenitor cells responsible for HO in FOP. Dramatic basic science discoveries of the biomolecules that are responsible for the emergent properties of FOP coupled with a comprehensive understanding of the natural history of FOP fueled the advent of clinical trials for FOP—a dazzling place to be for an ultra-rare condition that had existed in the backwaters of medicine for over three centuries [8,9,10,11,12,13,14,15,16,17].

In a transformative article entitled “*A New Grammar for Drug Discovery*”, Fishman and Porter argue that signaling pathways in cells provide the “right level” for drug discovery [18]. The current dynamic landscape of FOP research is firmly based on the signaling pathways and evolving targets that are being revealed at rapid speed.

This Special Issue of *Biomolecules*, “**Fibrodysplasia Ossificans Progressiva (FOP): From Molecular Mechanisms to Therapeutic Strategies**”, aims to explore the connection between molecular mechanisms and therapeutic strategies that are currently being explored in FOP, and by extension in more common disorders of HO that plague millions of individuals worldwide. This Special Issue of *Biomolecules* includes reviews as well as original research papers from experts in the field, providing the reader with advances in the understanding of FOP as well non-genetic forms of HO.

These articles encompass a broad range of topics that address the molecular genetics of FOP, developmental pathways in FOP, immunological aspects of FOP, molecular crosstalk in seminal pathways leading to HO in FOP, senescence-mediated pathways in FOP, progenitor cells that orchestrate HO in FOP, molecular imaging in FOP, gene therapy in FOP, pharmaceutical perspectives in FOP and non-genetic forms of HO.

Towler and colleagues comprehensively review the molecular biology of FOP and emphasize that missense mutations in the *ACVR1* gene lead to severe degenerative arthropathy and joint ankylosis independently of HO. Importantly, the authors indicate that in the same way that studying the genetic cause of HO has advanced our understanding of HO initiation and progression, insight into the roles of ACVR1 signaling during tissue development, particularly in the musculoskeletal system, can be gained from examining altered skeletal development in individuals with FOP. In addition, Towler and colleagues point out that ACVR1 is ubiquitously expressed in multiple tissues during development and may lead to a litany of less common, non-skeletal symptoms [19].

Progressive HO is the most dreaded complication of FOP and is stimulated by inflammatory triggers. In a comprehensive review of the immunological aspects of FOP, Diolintzi and colleagues explore the central and pivotal role of inflammatory mediators in HO suggesting that the immune system may be a common target for blocking HO in both FOP and non-genetic forms of HO [20].

Pignolo and colleagues describe cellular senescence as a critical hallmark of aging and explain how it is thought to be a tumor-suppressive phenomenon with characteristic features of irreversible growth arrest and apoptosis resistance, as well as a critical component of inflammation through the inflammatory senescence-associated secretory phenotype (SASP). Importantly, they review possible roles for cellular senescence in the inflammatory trigger and microenvironment of incipient HO and how targeting senescent cells may provide new therapeutic approaches to both FOP and acquired forms of HO [21].

Cellular hypoxia, a vital aspect of inflammation, promotes HO by amplifying BMP signaling [22,23]. In a comprehensive review, Wang and colleagues visit the recent progress on the mechanisms of the HIF-1α and mTOR pathways in the amplification of HO lesions in FOP and discuss strategies for the inhibition of HIF-1α and mTOR pathways in FOP as well as in non-genetic forms of HO [24].

Srinivasan and colleagues review the important role of Activin A as a therapeutic target in FOP and recount the discovery of Activin A as an obligate ligand of mutant ACVR1 [25,26,27]. They describe how the importance of Activin A in FOP led to a phase 2 clinical trial of an investigational antibody that blocks Activin A in patients with FOP [14]. They go on to review the role of Activin A in fibroadipogenic progenitors, the cells in muscle tissue that form HO, and the discovery of non-signaling complexes between Activin A and wild-type ACVR1 and their role in tempering HO in FOP mouse models [28,29].

Cong and Yang delve deeply into the biomolecular underworld and uncover the crucial role of the Yap–Ihh axis in the pathogenesis of FOP and suggest that the inhibition of Ihh or Yap may offer a potential therapeutic strategy to prevent and reduce HO in FOP and other genetic and sporadic forms of HO [30].

In an original article, Groppe and colleagues explore the structural–functional basis for the canonical gain of function ACVR1^R206H^ mutation in FOP. This detailed investigation into the three-dimensional modeling of ACVR1 offers new insights into the inhibition of mutant ACVR1 and avenues for novel treatment strategies [31].

In an original article, Burdick and colleagues present data that identify sex as a critical variable in translational and pre-clinical studies of FOP, and signal caution to investigators to consider sex in the design and interpretation of these studies [32].

Although plain radiography and computed tomography (CT) have been the mainstay of FOP clinical assessment and management, 18-fluor-sodium fluoride positron emission tomography/computed tomography ([^18^F]NaF PET/CT) was introduced recently as an experimental marker for ossifying FOP activity. In a review, Zwama and colleagues address the applicability of [^18^F]NaF PET/CT imaging as an investigational monitoring modality in FOP and associated disorders of HO [33].

Gene therapy has been proposed as a possible therapy in FOP and other monogenic disorders that affect the skeletal system. In an original article, Yang and colleagues explore the adeno-associated virus (AAV) as a promising delivery vehicle for gene therapy as well as a potential therapeutic strategy to explore in suppressing HO in FOP [34].

All forms of HO result in an irreversible loss of mobility and an incredible increase in morbidity. The keys that unlock the mysteries of FOP have unlocked and will continue to unlock the mysteries of the much more common and sporadic forms of HO. Juan and colleagues review a wealth of important advancements made in the study of both sporadic HO and FOP to better diagnose, comprehend, prevent, and treat both [35]. The keys to the closet are, in fact, the keys to the kingdom.

We hope that the readers will enjoy reading this Special Issue of *Biomolecules* and that the findings presented here will help advance the understanding of FOP as well as more common and sporadic forms of HO.

Frederick S. Kaplan, M.D.

Robert J. Pignolo, M.D., Ph.D.

Guest Editors
